# Supplementary material for: Translating synthetic natural language to database queries with a polyglot deep learning framework
Source: Sci Rep. 2021 Sep 16;11:18462. doi: 10.1038/s41598-021-98019-3 (PMC8445976; doi:10.1038/s41598-021-98019-3)
Supplement: Supplementary file 1 — Supplementary Information. [file 41598_2021_98019_MOESM1_ESM.pdf]

Supplementary Material for:

Translating synthetic natural language to database queries with a polyglot deep learning framework

by

Adrián Bazaga, Nupur Gunwant and Gos Micklem

show assignedby in hpoevidence
give confidence, description, name having primaryidentifier >= @value, type is @value in interactiondetail, interactionexperiment
from publication, give abstracttext, pages, issue with title lower than @value
what is primaryidentifier having secondaryidentifier >= @value from cds

Table S1: Examples of synthetic English queries generated during the random generation process.

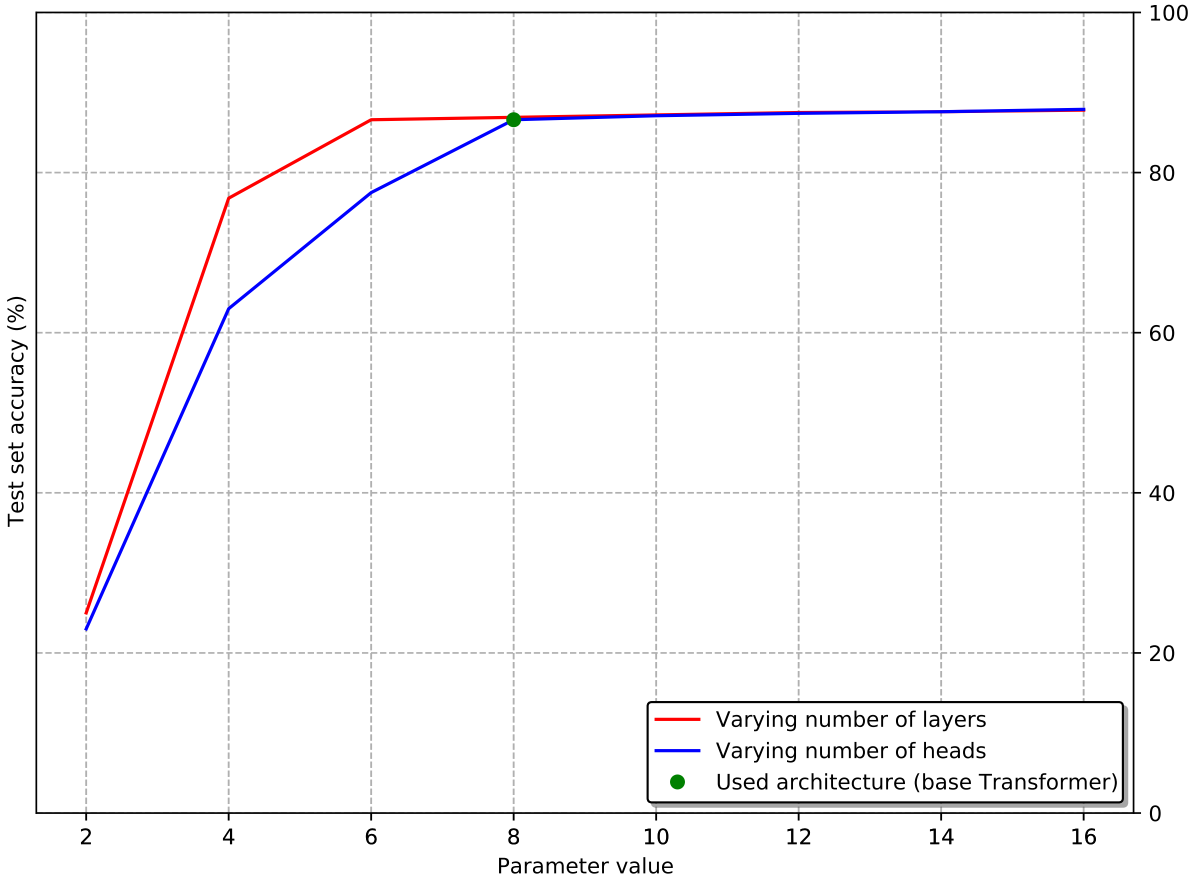

Figure S1: Ablation study for the Transformer model used in this paper, for HumanMine with N = 25000. Red line: test set performance for a varying number of network layers keeping the number of heads the same as the base Transformer (h = 8). Blue line: test set performance for a varying number of heads keeping the number of network layers the same as the base Transformer (L = 6). Green mark: Base transformer model, which was the selected architecture in this work.

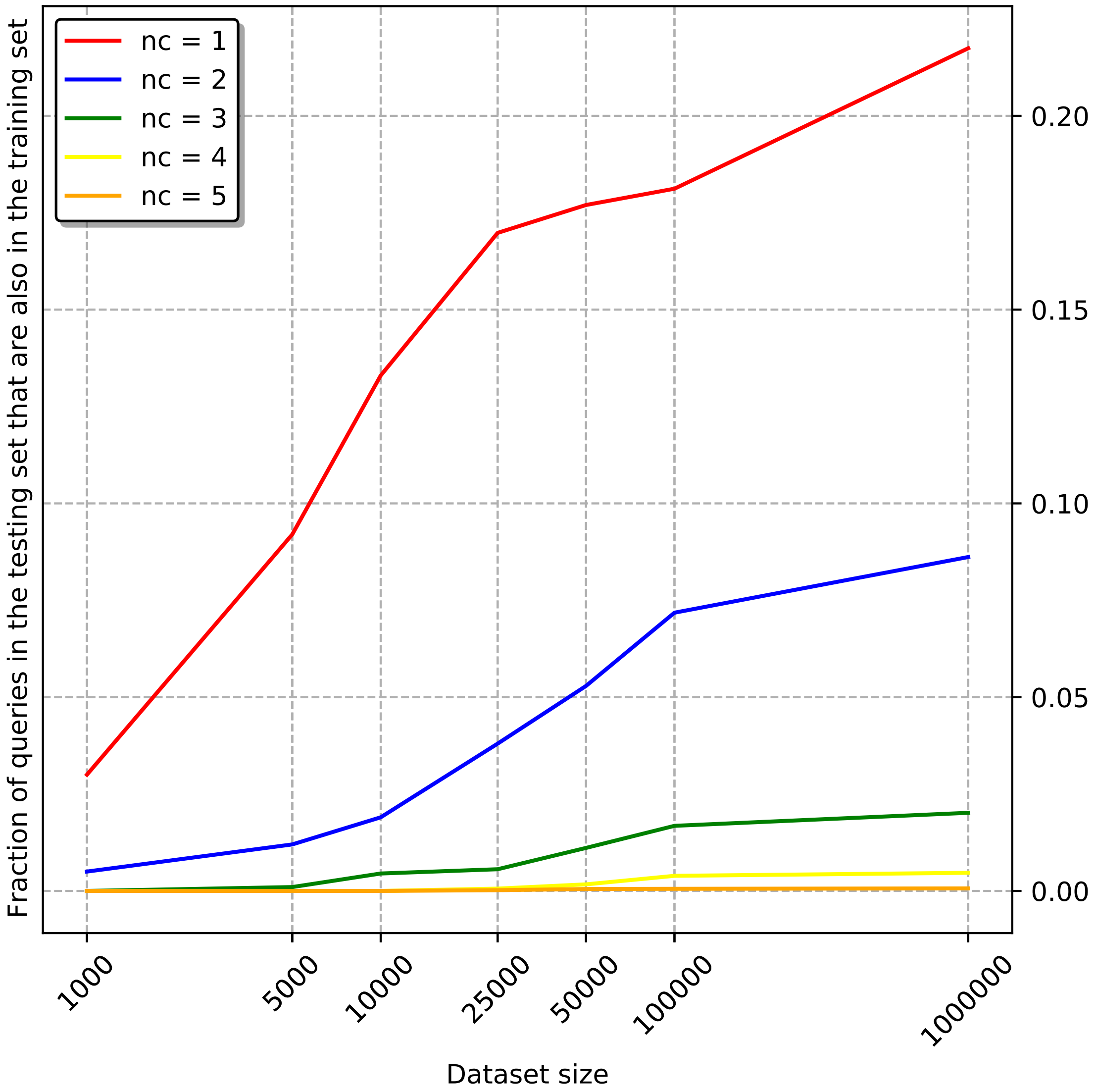

Figure S2: Overlap analysis: the HumanMine dataset was composed of equal numbers of queries covering 1-5 classes. We determined the degree of overlap between training and test sets by assessing the fraction of queries in the testing set that contained the exact same classes and attributes as a query or queries in the training set. This will be an overestimate of the true overlap as it does not take into account any constraints or constraint logic. Note that in fact nc = 5 was not used for training but the comparison is included here for completeness.

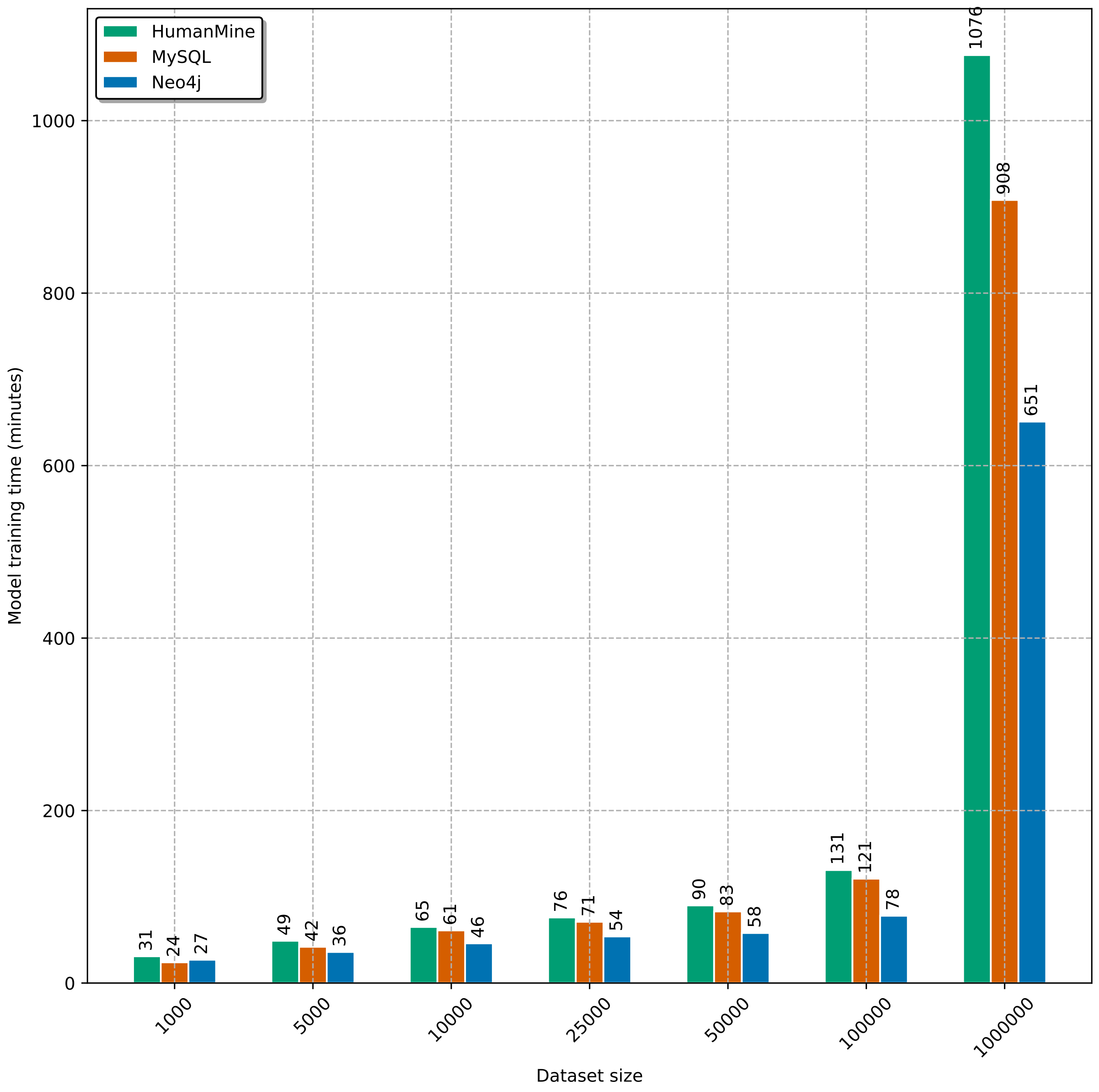

Figure S3: Model training times (in minutes) for increasing training dataset sizes across the three database systems tested. Training was carried out on a workstation with a single GPU (NVIDIA GeForce RTX 2070).
